# Individualized Gaussian process-based prediction and detection of local and global gray matter abnormalities in elderly subjects

**DOI:** 10.1016/j.neuroimage.2014.04.018

**Published:** 2014-08-15

**Authors:** G. Ziegler, G.R. Ridgway, R. Dahnke, C. Gaser, for The Alzheimer's Disease Neuroimaging Initiative

**Affiliations:** a Wellcome Trust Center for Neuroimaging, Institute of Neurology, London, UK; b Department of Psychiatry, Jena University Hospital, Jena, Germany; c Department of Neurology, Jena University Hospital, Jena, Germany

**Keywords:** Brain morphology, Lifespan brain aging, Gaussian processes, Single case analysis, Bayesian inference

## Abstract

Structural imaging based on MRI is an integral component of the clinical assessment of patients with potential dementia. We here propose an individualized Gaussian process-based inference scheme for clinical decision support in healthy and pathological aging elderly subjects using MRI. The approach aims at quantitative and transparent support for clinicians who aim to detect structural abnormalities in patients at risk of Alzheimer's disease or other types of dementia. Firstly, we introduce a generative model incorporating our knowledge about normative decline of local and global gray matter volume across the brain in elderly. By supposing smooth structural trajectories the models account for the general course of age-related structural decline as well as late-life accelerated loss. Considering healthy subjects' demography and global brain parameters as informative about normal brain aging variability affords individualized predictions in single cases. Using Gaussian process models as a normative reference, we predict new subjects' brain scans and quantify the local gray matter abnormalities in terms of Normative Probability Maps (NPM) and global z-scores. By integrating the observed expectation error and the predictive uncertainty, the local maps and global scores exploit the advantages of Bayesian inference for clinical decisions and provide a valuable extension of diagnostic information about pathological aging. We validate the approach in simulated data and real MRI data. We train the GP framework using 1238 healthy subjects with ages 18–94 years, and predict in 415 independent test subjects diagnosed as healthy controls, Mild Cognitive Impairment and Alzheimer's disease.

## Highlights

• We propose an approach to support individualized clinical decisions in elderlies.
• Gaussian process models are used to build a normative generative model of aging.
• It affords probabilistic predictions of local gray matter volume in subjects.
• We validate the model using simulated and large real MRI data samples.

## Introduction

Magnetic Resonance Imaging (MRI) and computational morphometry have become invaluable tools for in-vivo exploration of the underlying changes in healthy and pathological brain aging (Fjell and Walhovd, 2010, Frisoni et al., 2010). Consistent findings show that regional gray matter volume, as well as cortical thickness, exhibit substantial decline as a process of healthy aging (Fjell and Walhovd, 2010, Raz and Rodrigue, 2006). Importantly, studies observed considerable variability of age-related structural trajectories across brain regions and healthy elderly individuals (Raz et al., 2005, Raz et al., 2010, Walhovd et al., 2011). An open question in clinical practice still is, how to efficiently identify local pathological brain aging in individuals at risk of developing Alzheimer's disease (AD) or other types of dementia. Due to the large individual differences of normative age-related decline, the visual assessment of healthy vs. pathological local atrophy is a challenging task even for experienced radiologists. While single case studies are long-standing practice in neuropsychology (for overview of methods see e.g. Crawford and Garthwaite, 2012, McIntosh and Brooks, 2011), there is also an increasing number of neuroimaging studies using Voxel-based Morphometry (VBM) (Ashburner and Friston, 2000, Mechelli et al., 2005) that focus on single cases in comparison to a reasonably sized group of control subjects. These studies explored voxelwise macroanatomy in patients with neurological disorders like aphasia, Huntington disease, lesions, focal cortical dysplasia, epilepsy, cortical atrophy, and dementia (Colliot et al., 2006, Maguire et al., 2010, Mehta et al., 2003, Migliaccio et al., 2012, Mühlau et al., 2009, Mummery et al., 2000, Salmond et al., 2003, Scarpazza et al., 2013, Seghier et al., 2008, Sehm et al., 2011, Woermann et al., 1999).

In order to provide statistical measures of suspicious local brain volumes (or cognitive test scores) in single case studies, several parametric techniques have been proposed. A simple approach is to calculate z-scores using the control sample mean and standard deviation. If the observed z-score is found to be less than a certain percentile of the standard normal distribution, e.g. *z* < − 1.645 (corresponding to a one-tailed 95% percentile), the deviation might be considered statistically significant. Unfortunately, the z-score approach lacks the ability to account for the uncertainty of the control sample statistics, which might inflate type I errors especially in small samples (Crawford and Howell, 1998). Thus, the more conventional parametric approach to single case inference is the two sample t-test using a ‘pooled’ estimate of the variance (for details see e.g. Mühlau et al., 2009). The t-test statistic in the special case with *n* controls and one single patient reduces to $t=\left(\mu_{c}-\mu_{p}\right)/\left(\sigma_{c}\sqrt{1/n+1}\right)$ with control sample standard deviation *σ*_*c*_. Previous studies explored methodological issues using this type of unbalanced parametric design. In particular, small samples have been found to reduce sensitivity for detection of structural differences in single subjects (Mühlau et al., 2009). Unfortunately, for unbalanced designs the above difference score might be particularly affected by non-normality, rendering the t-test invalid (Salmond et al., 2002, Viviani et al., 2007). Robustness of the tests was found to be increased (type I errors reduced) by using larger smoothing kernels or appropriate transformations of the data. However, for inference in elderly subjects, the approaches often do not address the underlying developmental process, e.g. age-related effects in the control sample (see also Dukart et al., 2011), as well as variations due to other relevant covariates, e.g. global volume differences (Peelle et al., 2012).

Gaussian process (GP) models have emerged as a flexible and elegant approach for prediction of continuous, i.e. *y* ∈ ℝ, or binary, i.e. *y* ∈ [0, 1] variables (Kim and Ghahramani, 2006, Rasmussen, 1996, Rasmussen and Williams, 2006). Recently,GPs were successfully introduced to the neuroimaging community. The potential applications range from spatial priors (Groves et al., 2009), cortical maps (Macke et al., 2011), image denoising (Zhu et al., 2012), parameter estimation (Wang et al., 2012), white matter fiber clustering (Wassermann et al., 2010) and meta-analysis (Salimi-Khorshidi et al., 2011). GP models were shown to be particularly powerful for clinical applications, providing probabilistic predictions of symptom severity, pain states, recovery, cognitive and disease states using regression (Doyle et al., 2013a, Hope et al., 2013, Marquand et al., 2010) and classification (Hahn et al., 2011, Marquand et al., 2010, Mourao-Miranda et al., 2012, Pyka et al., 2012, Young et al., 2013) using functional and structural MR images as inputs. In addition to the common application as decoding or recognition models, i.e. making inference about causes of functional and structural brain states based on images (Friston et al., 2008), GPs might be particularly useful for generative modeling of individual differences of brain morphometry (see also Ashburner and Klöppel, 2011, Friston and Ashburner, 2004).

Here we propose a new approach to support individualized clinical decisions about an elderly patient's brain structure by providing quantitative, unbiased and highly transparent maps of local gray matter abnormalities and global volume z-scores for gray matter, white matter and cerebrospinal fluid. That means, the maps and z-scores aim at information support rather than providing fixed patient-level predictions about disease states derived from ‘black-box’ classifiers. GPs are used to implement a normative generative model of elderly subjects' local and global volumes in terms of a non-parametric function of subjects' covariates. The model captures normative age-related trajectories and effects of covariates typically observed in control samples. This implicitly assumes smooth structural trajectories without imposing strong constraints on the developmental model and thus allows more flexibility than low degree polynomial expansions (for discussion of quadratic fits see e.g. Fjell et al., 2010). At the same time it accounts for region specific late life accelerated gray matter shrinkage, which is shown to be part of healthy brain aging (Fjell et al., 2012, Fjell et al., 2013, Walhovd et al., 2011). The substantial individual differences of local and global volumes in elderly brains (i.e. even at the same age and fixed covariates) and the measurement noise are modeled in terms of Gaussian distributions and accounted for in individualized predictions. After model optimization in a large control sample, the local GP priors are conditioned on scans of new single subjects at risk of developing AD or other types of dementia. Training with a large pooled MRI database of 1238 healthy subjects with ages 18–94 years, and testing with an independent sample from the Alzheimer's Disease Neuroimaging Initiative dataset including subjects with MCI and AD, we show that the obtained normative probability maps (NPM) and global z-scores provide a powerful clinical application by quantitatively characterizing the single patient's abnormalities as compared to age-matched neurologically normal controls. This implements a Bayesian single case inference about structural abnormalities that flexibly accounts for predictive uncertainty in practical situations of different control data sample sizes, different data noise levels, and individual patient covariates, i.e. age, brain sizes, etc.

## Methods

### A Gaussian process model of cross-sectional gray matter observations in healthy elderly

Ideally, a generative model of the normative structural aging process accurately predicts the local gray matter volume *y* of an elderly study participant based on the age and a set of informative covariates **x** = [*age*, *sex*, …],i.e. forming a low dimensional covariate space $D\in {\mathbb{R}}^{m}$. The predictions require availability of most covariates for all cases in the training and test samples. Thereby, we here restrict our local generative model to six covariates summarized in **x**_*i*_ = [age, sex, tgmv, twmv, tcsf, fstr] for subject *i*, including demography and global parameters, i.e. total gray matter volume (tgmv), total white matter volume (twmv), and total cerebrospinal fluid (tcsf) obtained from MRI preprocessing. Furthermore, for inference about global atrophy an additional generative model for global brain parameters tgmv, twmv, and tcsf was applied using four covariates **x**_*i*_ = [age, sex, ticv, fstr] with ticv = tgmv + twmv + tcsf. Note, the proposed framework also naturally extends to physiological and behavioral factors, as well as subject independent but scan specific variables, e.g. the signal to noise ratio of the scan. In order to afford pooling across samples from 1.5 and 3 Tesla MRI scanners, we also included a field strength variable (fstr). The whole training sample covariate data is further denoted by **X**, which was obtained from concatenation of rows **x**_*i*_ for all *n* training subjects. The rows of brain data matrix **Y** (with entries *y*_*ij*_) refer to the GMV images of all *n* training subjects, and **y**_*j*_ is used to denote its *j*-th column, i.e. the data of voxel *j* from all subjects. Then the lifespan generative model of gray matter in voxel *j* follows

(1) $$\begin{array}{cc} y_{\mathrm{ij}}=g\left(x_{i},\theta_{j}\right)+\epsilon_{\mathrm{ij}}, & \epsilon_{\mathrm{ij}}∼N\left(0,\sigma_{j}^{2}\right) \end{array}$$

with subject index *i* and hyperparameter ***θ***_*j*_, an additive independent identically distributed Gaussian noise (also called the likelihood model) with variance *σ*_*j*_^2^. The latent (or noise free) variables *g*(**x**, ***θ***) incorporate our knowledge about aging and variability in different locations **x** of the covariate space D. We now exploit the function space perspective and define a GP prior, which implements our assumption about smoothness of the latent trajectories *g*(**x**, ***θ***). Technically, a GP is a distribution of functions, which is fully specified by its mean and its covariance function (for a technical introduction see Rasmussen and Williams, 2006)

(2) $$g∼\mathrm{GP}\left(m,\mathrm{cov}\right).$$

The following specification of the prior mean *m* and covariance function *cov* implies a distribution over latent structural trajectories and their individual differences in voxel *j*

(3) $$m\left(g\left(x_{p},\theta_{j}\right)\right)=0$$

(4) $$\mathrm{cov}\left(g\left(x_{p},\theta_{j}\right),g\left(x_{q},\theta_{j}\right)\right)=k_{\theta_{j}}\left(x_{p},x_{q}\right).$$

The main idea here is to suppose the covariance of the latent local and global volumes *g*(**x**, ***θ***_*j*_) to be a function of the similarities among subjects in covariate space D, expressed by a kernel mapping *k*. However, because the contribution of each dimension of D to latent variables *g* is a-priori unknown, we implement the kernel using a squared exponential function with automatic relevance determination (ARD) (Neal, 1996) for each voxel *j*

(5) $$k_{\theta_{j}}\left(x_{p},x_{q}\right)=a_{j}\exp\left(-\frac{1}{2}{\left(x_{p}-x_{q}\right)}^{T}\mathrm{diag}{\left(\ell_{j}\right)}^{-2}\left(x_{p}-x_{q}\right)\right),$$

with covariance hyperparameter ***θ***_*j*_ = (*a*_*j*_, ***ℓ***_*j*_), i.e. amplitude *a*_*j*_ and characteristic length scales ***ℓ***_*j*_ = [*ℓ*_1_^(*j*)^, …, *ℓ*_6_^(*j*)^] corresponding to axes of space D (similarly with 4 dimensions for global models). In particular, smaller values of length scale *ℓ*_1_^(*j*)^ indicate shorter timescales of lifespan developmental dynamics in voxels *j*. Intuitively, this parametrization of the kernel mapping symbolizes that either males or females with similar ages and global parameters are expected to have similar latent local gray matter volumes. Using the compact matrix notation the above model implies the following covariance for observed local gray matter volumes in voxel *j*

(6) $$\mathrm{Cov}\left(y_{j}\right)=K_{\theta_{j}}+\sigma_{j}^{2}I,$$

with **y**_*j*_ referring to a column vector of all observations in voxel *j*, $K_{\theta_{j}}\equiv K_{\theta_{j}}\left(X,X\right)$ denoting the evaluated kernel $k_{\theta_{j}}$ for all pairs of training points **X** using the covariance hyperparameters ***θ***_*j*_, and *σ*_*j*_^2^ again denotes the noise model variance. A more compact way to introduce the above model and the Gaussian process prior in Eqs. (1), (2), (3), (4) using conditionals is

(7) $$p\left(y_{j}|g_{j}\right)=N\left(g_{j}|\sigma_{j}^{2}I\right)$$

(8) $$p\left(g_{j}|X\right)=N\left(0,K_{\theta_{j}}\right).$$

We introduce the marginal likelihood by marginalization over the latent function values **g**_*j*_ using the likelihood *p*(**y**_*j*_|**g**_*j*_, **X**) and the prior *p*(**g**_*j*_|**X**)

(9) $$p\left(y_{j}|X\right)=\int p\left(y_{j}|g_{j},X\right)p\left(g_{j}|X\right)dg_{j}.$$

The logarithm of the GP prior term can be further evaluated

(10) $$\log\;p\left(g_{j}|X\right)=-\frac{1}{2}g_{j}^{T}K_{\theta_{j}}^{-1}g_{j}-\frac{1}{2}\log|K_{\theta_{j}}|-\frac{n}{2}\log2\pi .$$

The integration over Gaussian likelihood and prior can be performed analytically (for details Rasmussen and Williams, 2006) and reveals the log marginal likelihood (or evidence) for the lifespan generative model of voxel j

(11) $$\log\;p\left(y_{j}|X\right)=-\frac{1}{2}y_{j}^{T}{\left(K_{\theta_{j}}+\sigma_{j}^{2}I\right)}^{-1}y_{j}-\frac{1}{2}\log|K_{\theta_{j}}+\sigma_{j}^{2}I|-\frac{n}{2}\log2\pi .$$

Model optimization for local and global models is performed using the conjugate gradient descent of the marginal likelihood, which is supposed to optimally balance data-fit (term one in Eq. (11)) and model parsimony (term two). All applications using GP inference and prediction on MRI data in this paper were performed using Gaussian Process Regression and Classification Toolbox 3.4 (GPML, http://www.gaussian-process.-org/gpml/code/matlab/doc/in-dex.html).

### Normative probability maps and global z-scores

The above generative lifespan model of local and global volumes affords individualized predictions for untrained patients at risk of disease related abnormalities. We denote the optimized model parameters with ${\hat{\theta }}_{j}$ and ${\hat{\sigma }}_{j}^{2}$. The corresponding test sample covariates and brain images are supposed to be contained in rows of matrices **X**^∗^ and **Y**^∗^ respectively. For the purpose of predictions in a clinical decision setting, we first consider the joint distribution of already observed gray matter values **y**_*j*_ and latent variables of new test subjects **g**_*j*_^∗^, i.e.

(12) $$\left[\begin{array}{c} y_{j}\\ g_{j}^{*} \end{array}\right]∼N\left(0,\left[\begin{array}{cc} K_{{\hat{\theta }}_{j}}+{\hat{\sigma }}_{j}^{2}I & K_{{\hat{\theta }}_{j}}\left(X,X^{*}\right)\\ K_{{\hat{\theta }}_{j}}\left(X^{*},X\right) & K_{{\hat{\theta }}_{j}}\left(X^{*},X^{*}\right) \end{array}\right]\right).$$

We obtain the predictive distribution of local gray matter volume for the patient's latent variables (see also Rasmussen and Williams, 2006)

(13) $$\begin{array}{c} p\left(g_{j}^{*}|X,y_{j},X^{*},{\hat{\theta }}_{j},{\hat{\sigma }}_{j}^{2}\right)=N\left({\bar{g}}_{j}^{*},\mathrm{Cov}\left(g_{j}^{*}\right)\right),\;\mathrm{with}\\ {\bar{g}}_{j}^{*}=K_{{\hat{\theta }}_{j}}\left(X^{*},X\right){\left[K_{{\hat{\theta }}_{j}}+{\hat{\sigma }}_{j}^{2}I\right]}^{-1}y_{j}\\ \mathrm{Cov}\left(g_{j}^{*}\right)=K_{{\hat{\theta }}_{j}}\left(X^{*},X^{*}\right)-K_{{\hat{\theta }}_{j}}\left(X^{*},X\right){\left[K_{{\hat{\theta }}_{j}}+{\hat{\sigma }}_{j}^{2}I\right]}^{-1}K_{{\hat{\theta }}_{j}}\left(X,X^{*}\right). \end{array}$$

By adding the local noise variance ${\hat{\sigma }}_{j}^{2}$ to the latent predictive variance we arrive at the predictive distribution for observed local gray matter in the test sample, given the incorporated knowledge about healthy structural aging in the training sample

(14) $$p\left(y_{j}^{*}|X,y_{j},X^{*},{\hat{\theta }}_{j},{\hat{\sigma }}_{j}^{2}\right)=N\left({\bar{g}}_{j}^{*},\mathrm{Cov}\left(g_{j}^{*}\right)+{\hat{\sigma }}_{j}^{2}I\right).$$

To implement a Bayesian single case inference, we evaluate the z-scores of the predictive distribution

(15) $$\delta_{\mathrm{ij}}=\frac{y_{\mathrm{ij}}^{*}-{\bar{g}}_{\mathrm{ij}}^{*}}{u_{\mathrm{ij}}},$$

(16) $$u_{\mathrm{ij}}^{2}=k_{{\hat{\theta }}_{j}}\left(x_{i}^{*},x_{i}^{*}\right)-k_{{\hat{\theta }}_{j}}{\left(x_{i}^{*},X\right)}^{T}{\left(K_{{\hat{\theta }}_{j}}+{\hat{\sigma }}_{j}^{2}I\right)}^{-1}k_{{\hat{\theta }}_{j}}\left(x_{i}^{*},X\right)+{\hat{\sigma }}_{j}^{2}.$$

**Fig. 1:**
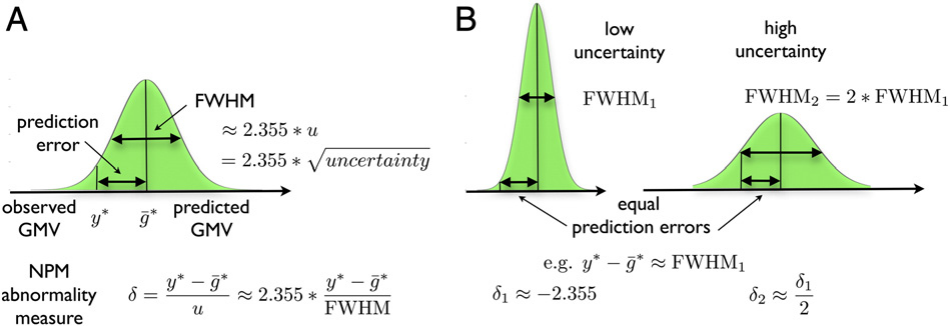
Illustration of the predictive distribution and core elements of the abnormality detection method. (A) The predictive distribution $p\left(y^{*}|X,y,x^{*},\hat{\theta },{\hat{\sigma }}^{2}\right)$ of an arbitrary voxel given the database covariates, database gray matter volumes, the test subject covariates, the optimized hyperparameters and local noise variance is shown in green. Its mode is the predicted gray matter volume ${\bar{g}}^{*}$ for the test subject. The difference of observed and predicted gray matter volume $y^{*}-{\bar{g}}^{*}$ we denote prediction error. The spread of the predictive distribution is called the predictive variance (or uncertainty) *u*^2^ (with *FW HM* ∝ *u*). The core of the method (and entries of NPMs) are abnormality measures *δ*, the z-scores, i.e. the prediction error in the unit of standard deviation of the predictive distribution. That is, *δ* evaluates the prediction error while accounting for differences of predictive uncertainty across subjects and voxels. (B) We illustrate that the NPMs appropriately account for uncertainty differences in all predictions. The prediction error $y-\bar{g}$ indicates the unexpected atrophy or hypertrophy under the generative model of normal aging. Then, observing the same prediction error in two subjects (or brain voxels) for instance with fourfold uncertainty (or equivalently twice FWHM) results in half of the value of *δ* and thus indicates less severe evidence for abnormality of the test subject.

The central ideas are illustrated in Fig. 1. The local z-scores *δ*_*ij*_ of test subject *i* at voxel *j* form the core of the proposed abnormality detection technique and will be denoted Normative Probability Maps (NPMs). The NPMs provide whole brain maps that reflect the probability to observe a particular patient's value (or even smaller values) of gray matter volume in a voxel, given the knowledge about structural lifespan development incorporated in the above generative model. The global z-scores were obtained analogous to the local NPMs. Strictly speaking, the exact probabilities would be obtained from evaluation of the cumulative predictive distribution. Nevertheless, this integration is expected to provide skewed distributions and thus might lack simplicity for practical clinical applications. Alternatively, we define the NPMs using z-scores, distributed around zero, with more negative values indicating stronger atrophy compared to normals and larger positive values showing hypertrophy respectively. The evaluation of local and global z-scores of the predictive probability densities sets the expected values for *δ*_*ij*_ for subjects drawn from the training population to zero. This additionally has the effect of normalizing the expected values across voxels. Note, that using NPMs for single case inference combines the prediction error, i.e. which here denotes the difference of observed and predicted volumes $y_{\mathrm{ij}}^{*}-{\bar{g}}_{\mathrm{ij}}^{*}$ (and which should not be confused with accuracy), and the predictive uncertainty *u*_*ij*_^2^, i.e. the variance of the predictive distribution. Intuitively, the advantage of such a Bayesian modeling approach for clinical decision support is that two predictions indicating the same prediction error (in the spirit of $y_{\mathrm{ij}}^{*}-{\bar{g}}_{\mathrm{ij}}^{*}$) can also be associated with very different levels of certainty or confidence (see Fig. 1B). NPMs and global z-scores account for these confidence differences and the model's predictions will strongly affect our conclusions for low compared to high predictive uncertainty. Inspecting the terms of the uncertainty (Eq. (16)) one can observe that it is increased by the prior variance and the noise term, and reduced by the information that the training sample provides about the test case.

### Clinical decisions based on NPMs

In contrast to common multivariate decoding schemes in dementia research (for review see Klöppel et al., 2012), our GP model affords inference about the local and global gray matter volume atrophy, which is an inference about the consequences rather than the cause, i.e. the disease status *D* = [*healthy*, *AD*, *LewyBody*, *etc*.] (see also Friston and Ashburner, 2004). However, we aim to show that NPMs and global z-scores might support the clinical decision process by providing a likelihood model for the implicit inference performed by the clinician. A reasonable inference about underlying disease states *D*_*i*_ would be obtained by applying Bayes-rule

(17) $$P\left(D_{i}|\mathrm{scan}\right)=\frac{P\left(\mathrm{scan}|D_{i}\right)P\left(D_{i}\right)}{P\left(\mathrm{scan}\right)}.$$

If we further make the simplifying assumption of conditional independence across voxels denoted with *y*_1_, …, *y*_*l*_

(18) $$P\left(\mathrm{scan}|D_{i}\right)=P\left(y_{1},\ldots ,y_{l}|D_{i}\right)\approx P\left(y_{1}|D_{i}\right)\ldots P\left(y_{l}|D_{i}\right)$$

we arrive at the approximate posterior distribution for disease states

(19) $$P\left(D_{i}|\mathrm{scan}\right)\approx \frac{P\left(D_{i}\right)}{P\left(\mathrm{scan}\right)}\prod_{j=1}^{l}P\left(y_{j}|D_{i}\right).$$

A clinician following a naive Bayesian inference about the unknown disease state of an individual with a brain scan (and gray matter volumes *y*) might implicitly apply mental representation of disease priors *P*(*D*_*i*_) (obtained from clinical experience) and the likelihood under different generative models for competing hypotheses *P*(*y*_*j*_|*D*_*i*_). We therefore assume that medical expert's decision can be supported by providing quantitative, valid, and transparent likelihood maps. The above introduced z-scores provide a measure of the likelihood of observing *y*_*j*_ under the generative model of healthy aging, i.e. *P*(*y*_*j*_|*D*_*i*_ = *healthy*), and thus might form a useful reference for all decisions about alternative disease states.

## Application to simulated data

In order to demonstrate the validity and potential of the proposed GP model, we used ground truth simulations followed by an application to real MRI data. The simulations were designed to emphasize two major purposes of the model outlined in the following two sections.

### Predictions of sparsely sampled individual developmental trajectories based on subject's covariates

**Fig. 2:**
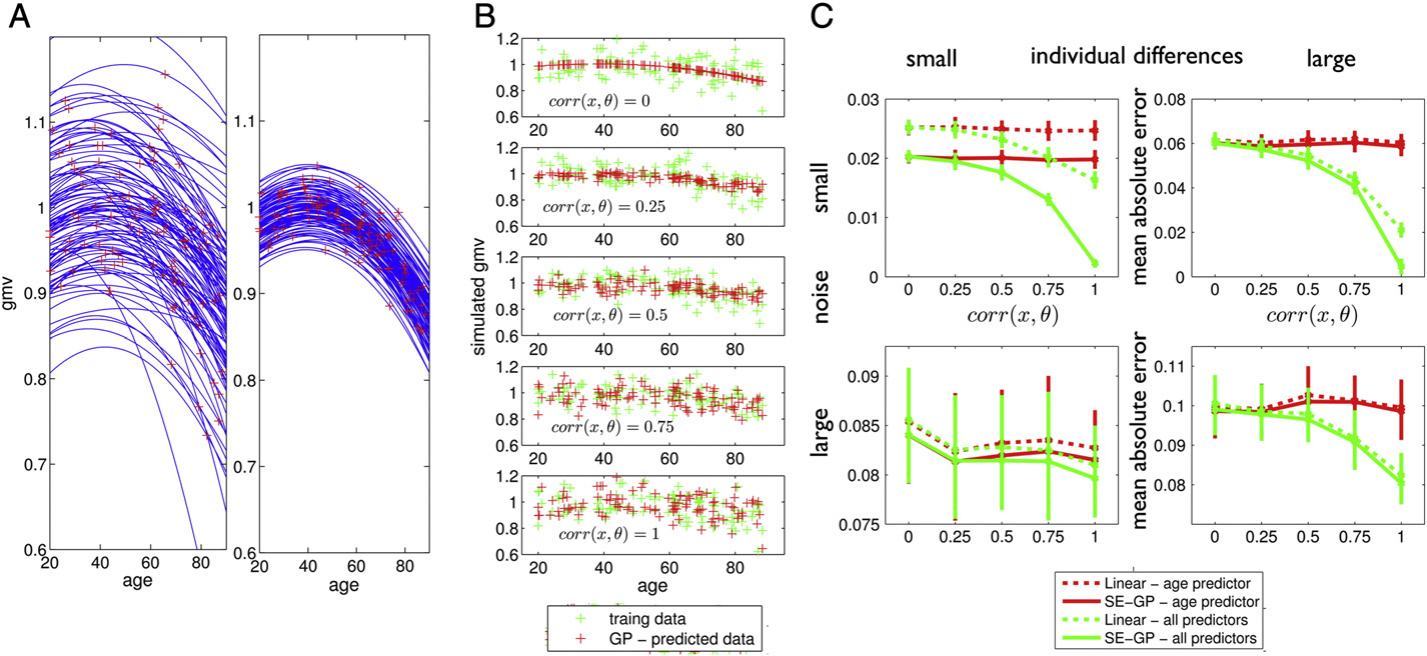
Simulation of structural trajectories, cross-sectional sampling and predictions based on correlates of individual change parameters. (A) Illustration of simulation of data *y*_*i*1_ for *i* = 1, …, 640 subjects using the mixed-effects modeling framework described in Eqs. (20), (21). Two random ensembles of individual trajectories using a quadratic model parametrization *f*(*t*_*ij*_, ***θ***_*i*_^(1)^) = *θ*_0*i*_ + *θ*_1*i*_*t*_*ij*_ + *θ*_2*i*_*t*_*ij*_^2^ with expectation of change parameters *E*(***θ***^(1)^) = ***θ***^(2)^ = [0.92, 4 ⋅ 10^− 3^, 1 ⋅ 10^− 4^] and either large individual differences (A left) with *Var*(***θ***^(1)^) = *diag*([6 ⋅ 10^− 3^, 1 ⋅ 10^− 6^, 2 ⋅ 10^− 10^]) or small individual differences (A right) with *Var*(***θ***^(1)^) = *diag*([6 ⋅ 10^− 4^, 1 ⋅ 10^− 7^, 2 ⋅ 10^− 11^]). Only 100 trajectories are shown. Red crosses indicate cross-sectional observations corresponding to a sparse sampling of trajectories with a single MRI observation per subject *i* at age *t*_*i*1_ distributed uniformly over the adult lifespan [20, 90]. Observations were performed with additive i.i.d. Gaussian noise with either *Var*(ϵ_*ij*_^(1)^) = 0.01 (large noise) or 6 ⋅ 10^− 6^ (small noise) respectively. Independent training and test ensembles were simulated. The second level model (Eq. (21)) also included random correlates/covariates **x**_*i*_ = [*x*_0*i*_, *x*_1*i*_, *x*_2*i*_] of ground truth individual change parameters ***θ***_*i*_ under variation of the correlation size to the true change parameter, i.e. *corr*(*x*_*k*._, *θ*_*k*._) = 0, 0.25, 0.5, 0.75, 1. (B) Gaussian process based predictions (using Eq. (14)) of observations *y*_*i*1_ based on subject covariates **x**_*i*_ after training in one ensemble and testing in a second independent ensemble. Rows illustrate the increase of precision of predictions under different correlation sizes *corr*(*x*, *θ*). (C) Mean absolute error of predictions for small and large amounts of noise (first level error) and individual differences (second level error) for a GP model with squared exponential covariance (‘SE-GP’) and predicting the same data using the general linear model (‘Linear’) estimated in the training data. Both, predictions using only subject's age and using all individual covariates, i.e. [*age*, *x*_0*i*_, *x*_1*i*_, *x*_2*i*_], indicate improvements in using the Gaussian process model for different types of data.

The GP model affords accurate between-subject level predictions for gray matter observations in samples with large individual differences and age-related effects. The predictions are made using a set of subject's covariates, e.g. age, demography, or other brain parameters. In order to realize simulations we here suppose (A) that gray matter observations in healthy development and aging essentially stem from sparse temporal sampling of an ensemble ℰ of individual trajectories and (B) that the considered covariates of interest are physiological or behavioral correlates or contributors to the variability within the ensemble. We formalize these assumptions by introducing the following two-level mixed-effects model of the ensemble of trajectories ℰ. The first level model is based on the assumption that the trajectory of underlying volumetric changes is sampled from subject specific functions of age or time

(20) $$y_{\mathrm{ij}}=f\left(t_{\mathrm{ij}},\theta_{i}^{\left(1\right)}\right)+\epsilon_{\mathrm{ij}}^{\left(1\right)}$$

where the measurement *y*_*ij*_ is the *j*-th observations obtained from the *i*-th subject at time (age) *t*_*ij*_, and ϵ_*ij*_^(1)^ denotes a Gaussian measurement error. In particular, we parameterize the trajectory using a quadratic polynomial expansion of age *f*(*t*_*ij*_, ***θ***_*i*_^(1)^) = *θ*_0*i*_ + *θ*_1*i*_*t*_*ij*_ + *θ*_2*i*_*t*_*ij*_^2^. This first level of the ensemble can be further summarized by **y** = *X*^(1)^***θ***^(1)^ + ϵ^(1)^. Although the true individual change parameters ***θ***_*i*_^(1)^ might be unknown, we here suppose to have access to their physiological or behavioral correlates, i.e. subject specific effects *X*_*c*_ = [*x*_1_, …, *x*_*r*_], e.g. demographic variables, or global brain parameters, etc. The contribution of these covariates to the first level change parameters is described by the second level model

(21) $$\theta^{\left(1\right)}=X^{\left(2\right)}\theta^{\left(2\right)}+\epsilon^{\left(2\right)}$$

with the parameters ***θ***^(2)^ and design matrix *X*^(2)^ containing three columns of ones for each *θ*_0*i*_, *θ*_1*i*_ and *θ*_2*i*_ and further columns for the covariates *X*_*c*_. Notably, Gaussian noise ***ϵ***^(2)^ adds further random individual differences to the ensemble of trajectories. The purpose of applying a nonparametric GP model in this context of mixed-effects models of development, i.e. assuming $f∼\mathrm{GP}\left(m,\mathrm{cov}\right)$, is that it affords predictions for measures of brain structure *y*_*ij*_ based on observations of individual covariates *X*_*c*_ without knowing the explicit parametrization of trajectory shape *f*(*t*, *θ*), the information about temporal sampling contained in *X*^(1)^, and the structure of the ensemble in design matrix *X*^(2)^. That means, GP model optimization corresponds to learning the functional form of a generative process. Fig. 2A depicts 100 trajectories of the simulated healthy subjects ensemble with either large (left) or small (right) individual differences. In order to obtain a typical cross-sectional sample of local gray matter volumes we assumed a single MRI scan per subject at random adult lifespan age (see red crosses in Fig. 2A). The observations *y*_*i*1_ were then modeled as a nonparametric GP function using subject's age and covariates of individual change parameters *x*_0*i*_, *x*_1*i*_ and *x*_2*i*_ as inputs, i.e. corresponding to *θ*_0*i*_, *θ*_1*i*_, and *θ*_2*i*_ respectively. The trained GP model allows predictions of observations in an independent ensemble of healthy subjects (Fig. 2B). We further varied the strength of correlation of accessible covariates and ground truth change parameters *corr*(*x*_*k*._, *θ*_*k*._) = 0, 0.25, 0.5, 0.75, and 1. As expected, we found that observing covariates that exhibit stronger relationships to the ground truth parameters of the mixed-effects generative process, affords better predictions in the cross-sectional sample. In order to compare the GP based predictions to existing methods, we additionally computed predictions for the test ensemble using general linear model (GLM) estimates in the ensemble used for training. Fig. 2C depicts mean absolute error of predictions in an independent test ensemble based on squared exponential covariance GP and GLM. Predictive performance was compared using either only subject's age or age together with three covariates *x*_0*i*_, *x*_1*i*_ and *x*_2*i*_. We independently varied the total amount of individual differences in terms of the second level error in the ensemble and the amount of noise in terms of the first level error. The obtained simulation for nonlinear trajectories suggests the advantages of GP based compared to GLM based predictions for different contexts of developmental data.

### Inference about local gray matter volume abnormalities in pathological aging trajectories

**Fig. 3:**
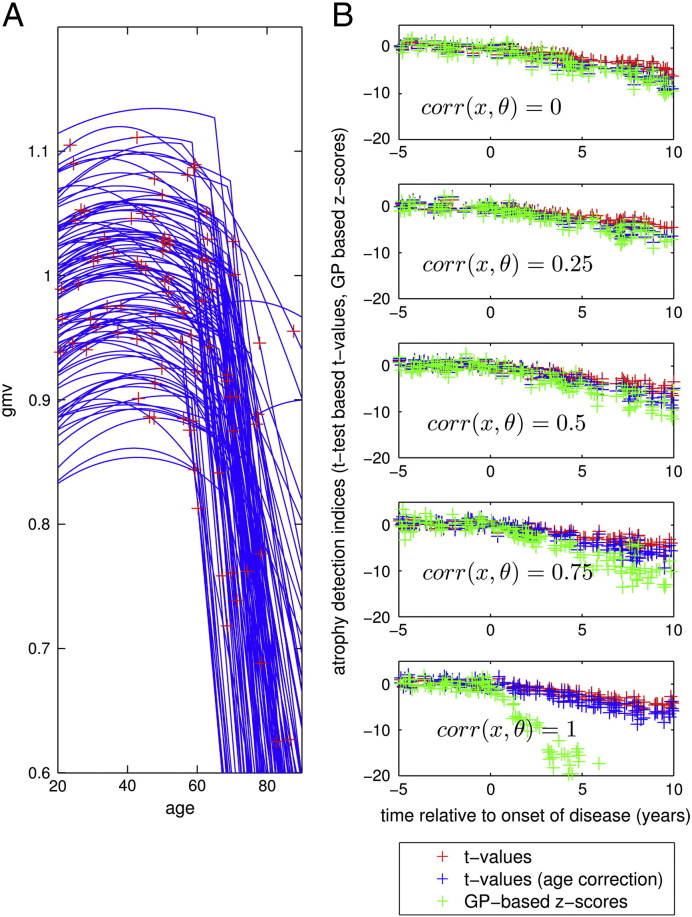
Simulation of an ensemble of structural trajectories with additive disease process and comparison of methods for their detection in an ensemble of healthy trajectories. (A) Illustration of simulations of observations *y*_*i*1_ for *i* = 1, …, 640 from diseased subjects using the mixed-effects modeling framework described in Eqs. (20), (21). The random ensemble of individual trajectories followed the healthy subject quadratic model with additional linear decline after individual age of onset at age *θ*_4*i*_: *f*(*t*_*ij*_, ***θ***_*i*_^(1)^) = *θ*_0*i*_ + *θ*_1*i*_*t*_*ij*_ + *θ*_2*i*_*t*_*ij*_^2^ + *θ*_3*i*_ ⋅ *max*([0, *t*_*ij*_ − *θ*_4*j*_]) with expectation of change parameters *E*(***θ***^(1)^) = ***θ***^(2)^ = [0.92, 4 ⋅ 10^− 3^, 1 ⋅ 10^− 4^, − 0.02, 65] and individual differences defined by *Var*(***θ***^(1)^) = *diag*[([6 ⋅ 10^− 3^, 1 ⋅ 10^− 6^, 2 ⋅ 10^− 10^, 1 ⋅ 10^− 4^, 20])]. Only 100 trajectories are shown. Red crosses indicate cross-sectional observations similar to the healthy ensemble. Observations were performed with additive i.i.d. Gaussian noise with noise *Var*(ϵ_*ij*_^(1)^) = 2 ⋅ 10^− 5^. (B) Comparison of different statistics for detection of increased atrophy in 640 healthy and 640 diseased test subjects using single case inference based on an independent healthy control sample. T-values $t=\left(\mu_{c}-\mu_{p}\right)/\left(\sigma_{c}\sqrt{1/n+1}\right)$ (see Introduction) are depicted in red over individual time of onset of disease. T-test after correction for age-effects in control and test sample is shown in blue. Proposed Gaussian process based z-scores (Eq. (15)) are shown in green. GP training is performed on an independent healthy ensemble of trajectories. Rows show different correlation sizes of the covariates (used for prediction with GP) with the true change parameter.

We further explored the potential of GP based inference about gray matter abnormalities in pathological aging. Having captured the large age-related variance and further individual differences in a healthy sample of brain development and aging, we here aim at evaluating the likelihood of unseen test subjects' brain scans given the database as a normative reference. We therefore simulated an ensemble of trajectories from diseased subjects ℰ_*d*_ by assuming a substantial additive linear disease process beginning at a random age of onset *θ*_4*i*_ : *f*(*t*_*ij*_, ***θ***_*i*_^(1)^) = *θ*_0*i*_ + *θ*_1*i*_*t*_*ij*_ + *θ*_2*i*_*t*_*ij*_^2^ + *θ*_3*i*_ ⋅ *max*([0, *t*_*ij*_ − *θ*_4*j*_]). With exception of this disease process, this parametrization of the trajectories is assumed to be identical to the ensemble of healthy subjects ℰ. Fig. 3A depicts 100 trajectories of simulated diseased subjects and the random age of MRI acquisition. In this context of mixed-effects models for development and aging, early disease detection corresponds to classifying new subjects based on a single observation into ensembles ℰ and ℰ_*d*_ respectively. As a proof of principle, we here compared the GP-based z-scores and t-values with respect to their ability to detect the abnormality of diseased subjects after the age of onset. Fig. 3B compares z-scores, and t-values, and t-values with correction of age effects in the control and test sample as a function of years relative to the individual onset of disease. We found that all considered indices exhibit comparable values before the age of onset. However, after disease onset the z-scores showed a steeper decline with years after onset compared to t-tests. This indicates an increased sensitivity for early disease detection, in particular using more informative covariates with higher correlation to the ground truth individual differences of the generative process. Based on the above assumptions, this finding indicates the potential of using informed predictions to access deviations from normality in real MRI data applications.

## Application to real MRI data

### Database

The GP models of normative aging were trained using a large cross-sectional healthy aging brain database, which has been pooled using healthy controls' T1-weighted MRI scans from four freely available multi-center samples. Firstly, we included 116 healthy controls' (ages 60–90 years, mean age 75.9 years) baseline/screening scans from the ADNI1 dataset of the Alzheimer's Disease Neuroimaging Initiative (ADNI, http://www.adni-info.org)^2^ (see also Mueller et al., 2005). Secondly, 316 healthy controls (ages 18–94, mean age 45.1 years) from the cross-sectional release of the Open Access Series of Imaging Studies (OASIS, http://www.oasis-brains.org) entered the database (Marcus et al., 2007). Thirdly, 561 subjects (19–85 years, mean age 48.0 years) from the IXI database (http://biomedic.doc.ic.ac.-uk/brain-development) were included. Finally, 245 participants (19–85 years, mean age 44.5 years) from the International Neuroimaging Data-sharing Initiative (INDI, http://fcon_1000.projects.nitric.ord/indi/indi_ack.html) Functional Connectome Project/INDI imaging sites Atlanta, Baltimore, Berlin, Cambridge, ICBM, Leiden, Milwaukee, Muenchen, and New-York entered the training sample (for additional information see Mennes et al., 2013). The sample selection of healthy elderly subjects was performed in order to realize a sufficient coverage of the adult lifespan age range. Apart from the chronological age, we did not apply any additional phenotypic information-based inclusion criteria. After excluding subjects with artifacts or errors during the MRI preprocessing steps (see section below) and pooling the subsamples we obtained the final VBM database for subsequent GP model training (n = 1238, 686/552 female/male, ages 18–94 years, mean 49.7, std 19.7). Notably, 245 subjects from the INDI sample and 180 subjects from the IXI sample were scanned with 3 Tesla scanners, while all other subjects in training and testing were scanned with 1.5 Tesla scanners. The differences due to variations of scanner field strength were explicitly accounted for in the subsequent modeling steps. The database samples densely over the adult lifespan containing 288/175/164/190/169/188/64 subjects with ages 18–30, 31–40, 41–50, 51–60, 61–70, 71–80, and 81–94 years respectively. Furthermore, we chose a large subsample of the ADNI1 database with T1-weighted scans of 415 study participants with ages 55–93 years to detect local brain abnormalities. This independent test sample contained 100 baseline scans of healthy subjects, 95 with a stable diagnosis of MCI during the whole ADNI study (sMCI), 92 converting from original MCI diagnosis at baseline to AD during the ADNI study (pMCI), and 128 scans of patients diagnosed with AD. Note, that we chose random non-overlapping subsamples of the ADNI healthy subjects for training and testing. This affords valid testing of the generalization capability of our approach.

### Image preprocessing

A detailed overview of the acquisition protocols can be found on the corresponding project references. From the available samples we included T1-weighted images with a maximum voxel dimension of 1.5 mm. All further preprocessing steps were performed inSPM8 (Wellcome Trust Centre for Neuroimaging, London, UK, http://www.fil.ion.ucl.ac.uk/spm) using the VBM8 toolbox (http://dbm.neu-ro.uni-jena.e/vbm). During preprocessing all images were interpolated to an isotropic resolution of 1.5 mm. The images were (1) corrected for bias-field inhomogeneities, (2) registered using a linear (i.e. 12-parameter affine) and a nonlinear diffeomorphic transformation (Ashburner, 2007), and (3) stripped of non-brain tissue in the T1-weighted images. Thereafter, some results from the SPM8 unified segmentation package (Ashburner and Friston, 2005) were used to initialize a VBM8 algorithm that classifies brain tissue into gray matter (GM), white matter (WM), and cerebrospinal fluid (CSF). In order to avoid introducing a systematic bias into the segmentation of adult and elderly subjects' brains the applied segmentation is prior free. The VBM8 segmentation contains partial volume estimation (PVE) to account for mixed voxels with two tissue types (Tohka et al., 2004). The algorithm uses an adaptive maximum a posteriori (AMAP) approach (Rajapakse et al., 1997) and a subsequent application of a hidden Markov random field model (Cuadra et al., 2005). Within the AMAP estimation, the local variations of the parameters (means and variance) are modeled as slowly varying spatial functions. This accounts for intensity inhomogeneities and other local variations. We further quality checked the database using covariance-based inhomogeneity measures of the sample as implemented in the VBM8 toolbox. Thereafter, the resulting gray matter volume images were multiplied voxelwise by the determinants of Jacobian matrices from SPM's nonlinear transformations. This modulation is done to adjust for local volume changes introduced by the nonlinear normalization. Finally, in order to explore the effects of different degrees of smoothing we reran all GP models using Gaussian kernels of 4, 8, and 12 mm full width at half maximum (FWHM) respectively. The images were masked by a binary image indicating voxelwise sample mean of gray matter volume exceeding absolute threshold of 0.05. All GP modeling steps were performed on subsamples of database images obtained using the above steps. To reduce computational expense the local GP optimization and predictive map predictions were performed in a downsampled 3 mm grid obtained from the 7th degree B-spline interpolation. The obtained 52,252 gray matter voxels from 1238 subjects were assumed to reflect aging-related differences, as well as normative individual variability in terms of fine-grained maps of local gray matter volume (GMV) content.

### Transformation of the data

Recent work on Voxel-based Morphometry methods has explored conditions, under which parametric tests may reveal invalid conclusions (Viviani et al., 2007). In particular, the authors showed that severe departures from normality of local gray matter volume distributions may affect significance thresholds, especially for highly unbalanced designs. Although classical frequentist and Bayesian inference schemes are fundamentally different, similar violations of the normality assumptions might introduce biases in our model estimates. As suggested by Viviani et al. (2007), applying heterogeneous voxel-by-voxel transformations might reduce non-normality and its consequences. We follow a similar approach by entering the preprocessed data to a voxelwise Box–Cox power transformation (Box and Cox, 1964) of the following form

(22) $$f_{\lambda }\left(y\right)=\left\{\begin{array}{cc} \begin{array}{l} \frac{y^{\lambda }-1}{\lambda }\\ \log\left(y\right) \end{array} & \begin{array}{l} \mathrm{if}\;\lambda \neq 0\\ \mathrm{if}\;\lambda =0. \end{array} \end{array}\right)$$

The local parameter *λ* for each voxel was chosen by maximization of the log-likelihood function

(23) $$L\left(\lambda \right)=-\frac{n}{2}\log\sigma_{\lambda }^{2}+\log J_{\lambda },$$

with the number of training samples *n*, the estimated residual variance *σ*_*λ*_^2^ under the maximum likelihood fit of the transformed data, and the Jacobian of the transformed data *J*_*λ*_. In order to preserve the voxelwise scaling of the transformed data for further modeling steps, we normalized the transformed data according to *f*_*λ*_. Due to the nonlinearity of mapping *f*_*λ*_ approximate normalization can be achieved using linear Taylor expansion around the mean *μ*, i.e. *f*_*λ*_(*y*) ≈ *f*_*λ*_(*μ*) + *f*_*λ*_′(*μ*)(*y* − *μ*), from which follows that *Var*(*y*) ≈ *Var*(*f*_*λ*_(*y*)/*f*_*λ*_′(*μ*)). Note, the local data transformation is a separate modeling step performed before subsequent GP modeling steps. This local parameter *λ* was determined using only the training database and further reused to transform the testing sample images in a similar way.

### Results

**Fig. 4:**
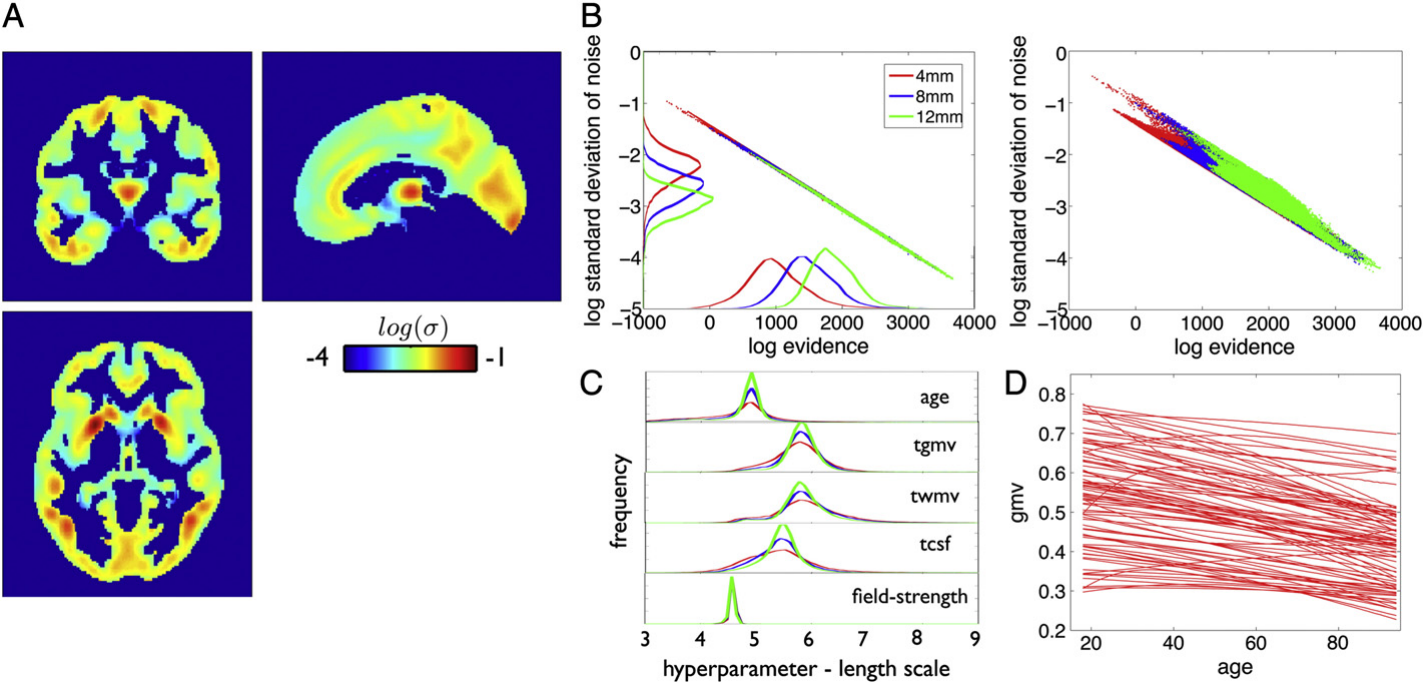
Gaussian process generative model of local gray matter volume using 1238 healthy adult subjects with ages 18–94 years. (A) The estimated local noise term $\log\left(\hat{\sigma }\right)$ of the GP model for data after smoothing with 8 mm Gaussian kernel is shown. The noise term captures unexplained variability of gray matter volume observations. Red and blue indicate larger vs. smaller local noise variance, respectively. (B) Shown is the relationship of local model evidence and the noise term $\log\left(\hat{\sigma }\right)$ (log log plot) across all voxels for different degrees of smoothing (left). In particular, by applying Gaussian kernels of 4 (red), 8 (blue), and 12 (green) mm FWHM. Additionally histograms of the corresponding indices are shown. The relationship of local model evidence and the standard deviation of gray matter volume observations (log log plot) across all voxels is shown for different degrees of smoothing (right). (C) Histogram of voxelwise length scale parameters *l*_1_^(*j*)^ of the input variables obtained from model optimization. (D) 100 random voxel lifespan gray matter volume trajectories of the estimated generative model. Shown is *g*(*x*, ***θ***_*j*_) as a function of subject's age.

In order to demonstrate the validity of the proposed GP framework for local and global abnormality detection in neuroimaging data, we trained the above specified models using the large healthy subject database. The noise variance captures the remaining variance in the observations unexplained by variability in covariate space. Fig. 4A depicts the obtained spatial pattern of the noise from data smoothed with a Gaussian kernel of 8 mm FWHM. With the exception of the thalamus, most cortical gray matter regions exhibited reasonably small noise variance. At the same time the evidence closely resembled the spatial pattern of the noise term, with higher evidence in regions with less unexplained variance in observations (Fig. 4B, left). Increased spatial smoothness in the observations reduced the local amount of noise and increased the model evidence. At least in part, this might be related to regional variance differences of modulated local gray matter volume after between-subject normalization (Fig. 4B right plot). Histograms of whole brain voxelwise characteristic length scales are shown in Fig. 4C. The proposed model is symmetric with respect to the dimensions of the covariate space and a projection on the age dimension reveals adult lifespan local structural trajectories (Fig. 4D).

**Fig. 5:**
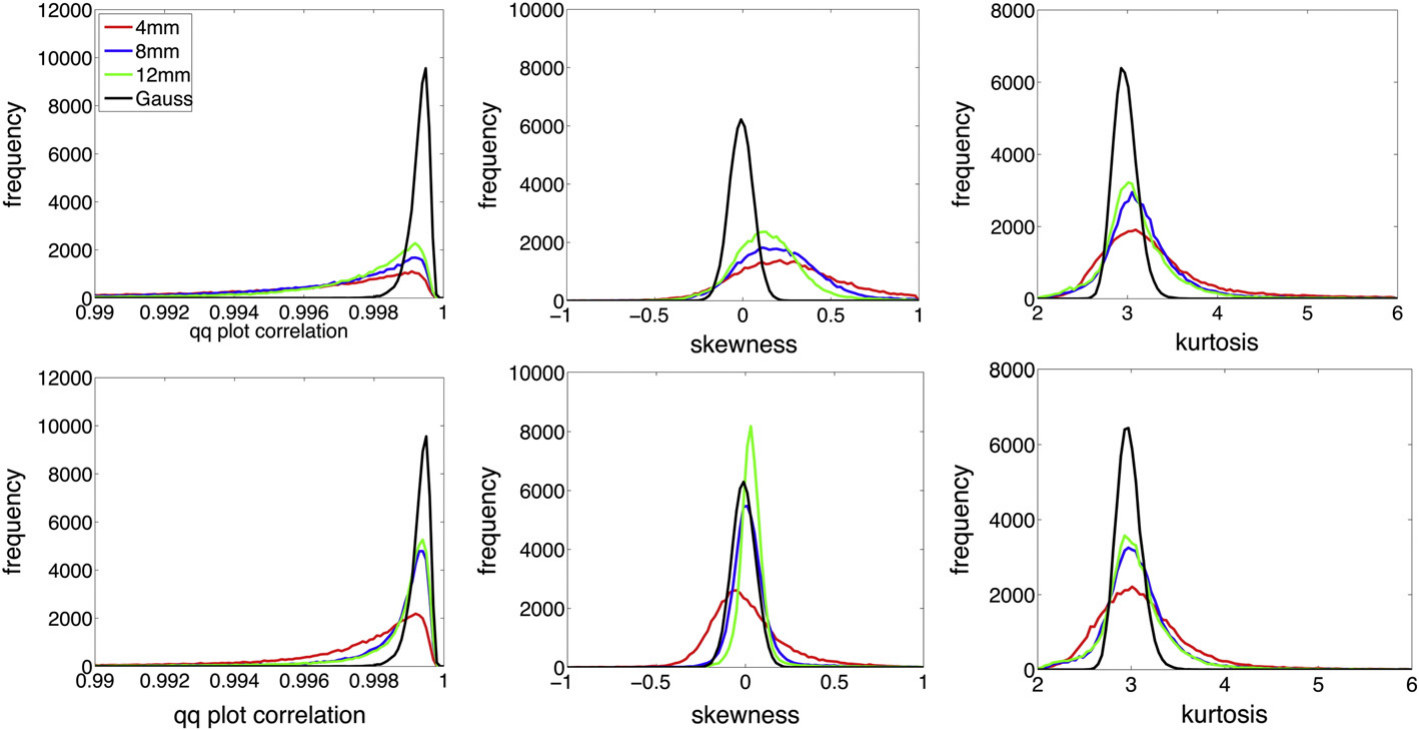
Gaussianity of the residuals under the generative model of local gray matter volume using 1238 healthy adult subjects with ages 18–94 years and different degrees of smoothing, in particular applying Gaussian kernels of 4 (red),8 (blue), and 12 (green) mm full width at half maximum (FWHM). Expected results under Gaussian assumptions are given in black. Top row shows residuals from untransformed data whereas the bottom row depicts voxelwise Box–Cox transformed data. (left) Histogram of Q–Q plot correlations of all voxels. (middle) Histogram of voxelwise sample estimates of skewness. (right) Histogram of voxelwise sample estimates of kurtosis.

An assumption that affords computationally tractable and efficient local GP model inference is the Gaussianity of the noise model. Moreover, any violations of Gaussianity might result in biased single case predictions and inference. As suggested by Ashburner and Friston (2000) the quantile–quantile (Q–Q) plot might provide a normality statistic for the model residuals. The Q–Q plots sample quantile versus the sample quantile that would be expected if the residuals were normally distributed. If so, a Q–Q plot would result in a straight line. A deviation from a straight line can be identified by calculating the correlation coefficient of the Q–Q plot. The expected and observed Q–Q plot correlation coefficients for our database are shown in Fig. 5A (left). The correlations were mainly observed in the interval [0.99, 1] but nevertheless exhibited slight deviations from the theoretical expectation under normality. We explored these deviations by calculating the third and fourth standardized moments of residuals, i.e. skewness and kurtosis respectively. As expected for modulated gray matter volume data, the residuals show a slightly positive skewness for many voxels (see Fig. 5A middle). Additionally, but less emphasized, kurtosis was found to be slightly higher than three indicating a more peaky distribution with heavier tails (see Fig. 5A right). Application of larger smoothing kernel sizes improved normality but left a noticeable positively skewed distribution of observations, even with large smoothing kernels. As recently suggested by Viviani et al. (2007) we explored the benefits of local Box–Cox transformation of the gray matter volume observations before subsequent GP modeling. Fig. 5B depicts Q–Q plot correlations and standardized moments of the model residuals after the transformation. Applying Box–Cox transform to the data substantially improved the Gaussianity by reducing residuals' skewness toward the expectations under normality assumptions.

**Fig. 6:**
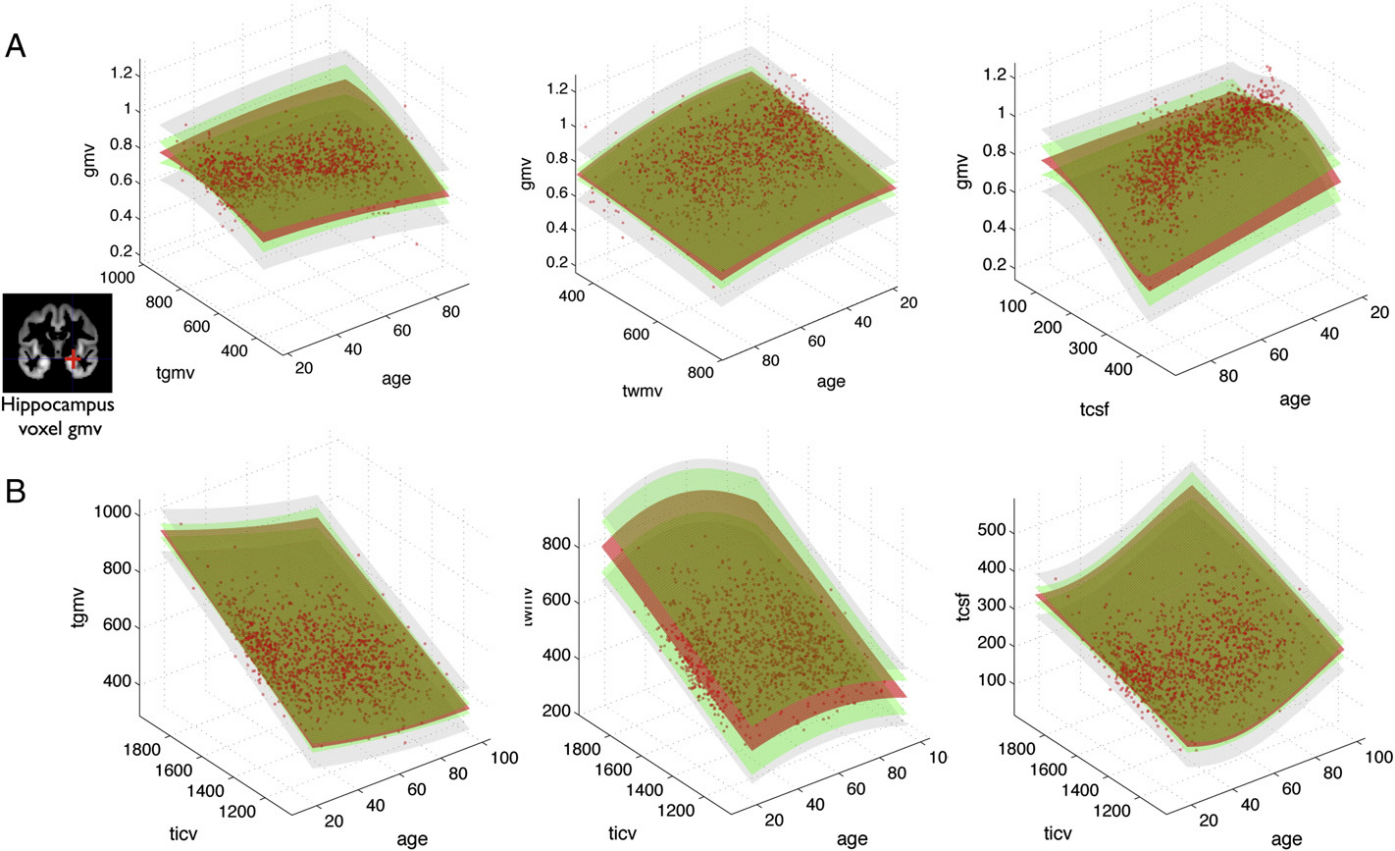
Gaussian process models of local and global gray matter volume in the 1238 healthy subject sample. (A) Illustrates the GP based generative model of local GMV in a single voxel in hippocampus (24, − 12, − 18) mm MNI. Columns indicate the dependency on age and total gray matter volume (tgmv, left) total white matter volume (twmv, middle) and total cerebrospinal fluid volume (tcsf, right) respectively. Depicted are 3D surface plots of model components, continuously shown over test locations in input dimensions. Posterior distribution of latent variables $g\left(x,\hat{\theta }\right)$ with expectation $\bar{g}$ (red) and standard deviation $\bar{g}\pm 1.96\mathrm{std}\left(g\right)$ (green) is shown. The likelihood model standard deviations *σ* = *std*(ϵ) is shown at $\bar{g}\pm 1.96\sigma$ (gray). (B) Illustrates the GP based generative model of tgmv (left), twmv (middle) and tcsf (right). Columns indicate total intracranial volume (ticv) and age respectively.

**Fig. 7:**
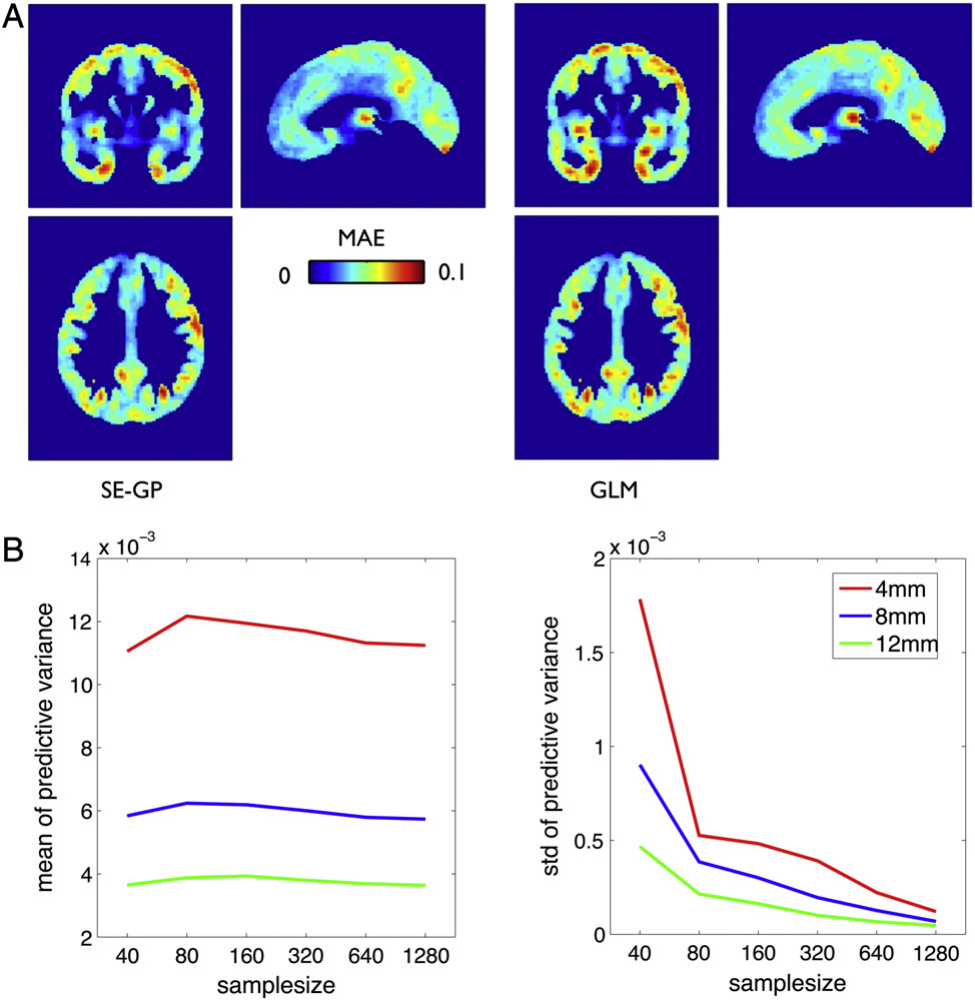
Gaussian process model predictions in independent ADNI test sample of healthy subjects. (A) Mean absolute error of local predictions using squared exponential GP (left) based on subject's age, sex, global volumes, and type of scanner as input variables. For method comparison the predictions were repeated using the general linear model (GLM) estimates *B* = (*X*′ ∗ *X*)^− 1^*X*′ ∗ *Y* of the all covariates *X* in the training sample for predictions *Yt* = *Xt* ∗ *B* in the test sample. (B) Exploring the effects of training sample size on the predictive variance (uncertainty) *u*_*ij*_^2^ for predictions of hippocampal GMV at (24, − 12, − 18) of the independent ADNI test sample. Random training subsamples of sizes 40, 80, 160, 320, 640 and 1238 were drawn and Gaussian kernels of 4 (red), 8 (blue), and 12 (green) mm full width at half maximum (FWHM) were applied. The local GP model optimization and ADNI healthy test sample predictions were repeated 20 times and the following parameters were averaged across these repetitions. The plots show the mean (left) and standard deviation (right) of the predictive uncertainty in the test sample. Increasing the training sample size mainly reduces the subject by subject variability of individual predictive uncertainty of the model.

Fig. 6 illustrates the single hippocampus voxel (at 24, − 12, − 18 MNI) local model (top row) and the three global models (bottom row) for each tissue class. In order to afford visualization, we only show the dependency on age and global parameters (top row) and on age and total intracranial volume (bottom row) using 3D surface plots. Models of local and global brain parameters indicate that GPs are able to capture nonlinear dependencies in the data. The core of GP model is the full posterior distribution of predictive latent variables, which also provides an uncertainty for all locations in the input space. Notably, we observed a profound effect of variation of training sample size that indicated an increased predictive latent uncertainty in sparsely sampled locations of the covariate space in smaller samples, e.g. inspecting very old people, very large brains etc. We further aimed at prediction of local gray matter volume in the independent ADNI test sample of 100 healthy subjects. Fig. 7A shows the mean absolute error (MAE) of local predictions using GPs (left) in direct comparison to the general linear model (right). The MAE was found to be smaller using the GP model, especially in temporal and medial temporal lobe gray matter regions. In addition to the prediction error, an integral part of the proposed method is the predictive uncertainty. Thus, we also explored the effects of training sample size and data smoothness on predictive uncertainty. We found that the average uncertainty in test sample was rather independent of sample size, and that image smoothness induced noise differences had stronger effects (see Fig. 7B left). However, the predictive uncertainty for single case decisions strongly varied across subjects in the test sample when training GP models with smaller control samples (see Fig. 7B right).

**Fig. 8:**
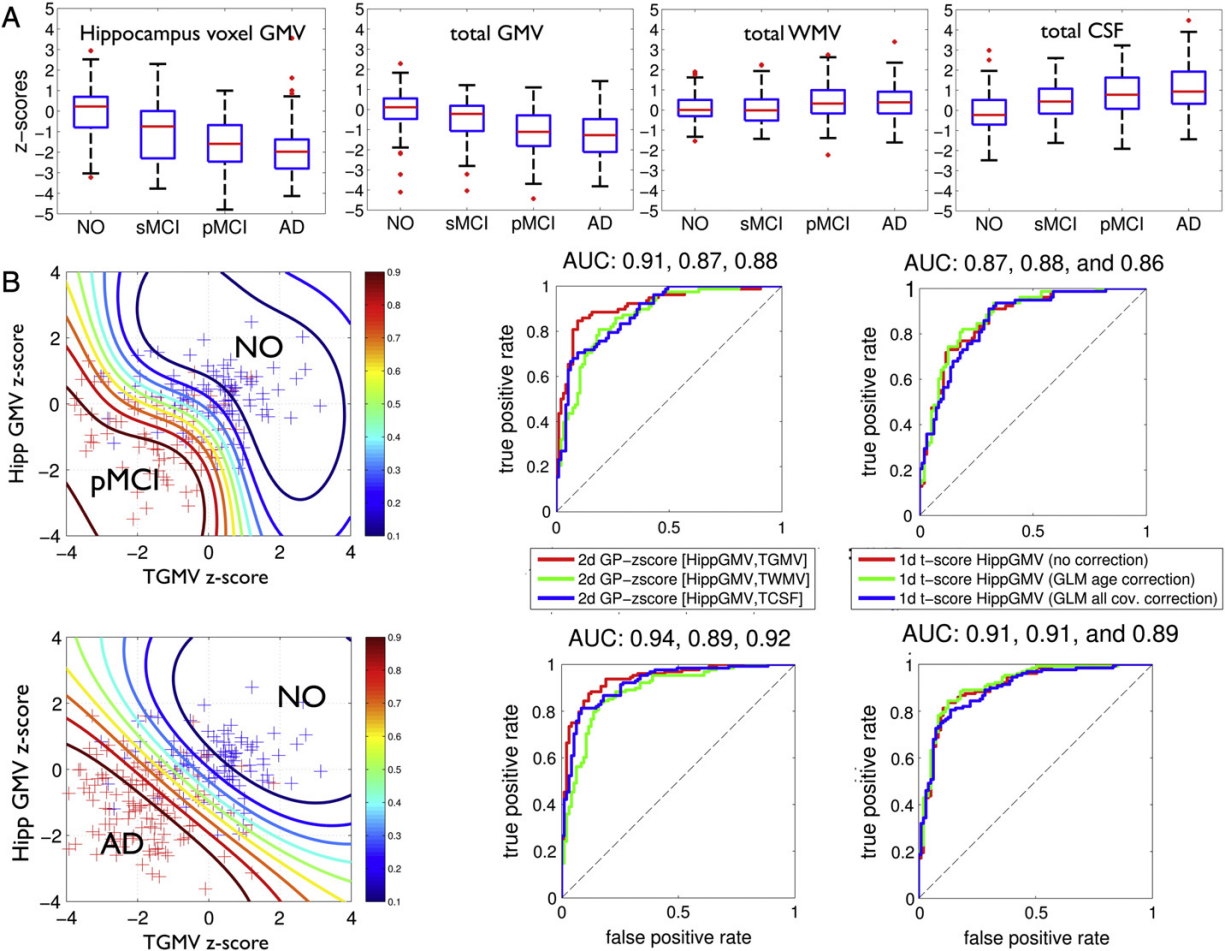
Gaussian process model based z-scores of global and local volumes in the ADNI test sample. (A) First column shows z-scores of predictive probabilities *δ*_*ij*_ of hippocampal voxel GMV at (24, − 12, − 18) mm MNI of 415 scans from study participants with ages 55–93 years. Separate boxplots for 100 NO, 95 stable MCI (sMCI), 92 progressive MCI (pMCI), and 128 AD subjects subgroups are shown. Columns 2–4 depict z-scores for the GP model of global volumes. (B) Potential of GP-based local and global volume z-scores and comparison to t-test based single case inference. The left column shows a characterization of the pMCI and NO (top) and AD and NO (bottom) subjects in 2 dimensional plot using z-scores from a single hippocampus voxel GMV and total gray matter volume respectively. Additional contours show predictive probabilities obtained by post hoc Gaussian process classification of the clinical vs. normal subjects using a squared exponential covariance and cumulative Gaussian likelihood function. Middle column shows receiver operating characteristic (ROC) curves of classification of pMCI vs. NO (top) and AD vs. NO (bottom) using a 2D Gaussian process classification with leave one out cross-validation. Colors indicate 2D classification based on local gray matter volume with tgmv (red), twmv (blue), and tcsf (green) respectively. The right column shows the same group classification based on 1d Gaussian process classification of uncorrected t-values (red), t-values after age correction using general linear model (GLM) (blue), and t-values after GLM based correction of all covariate effects.

**Fig. 9:**
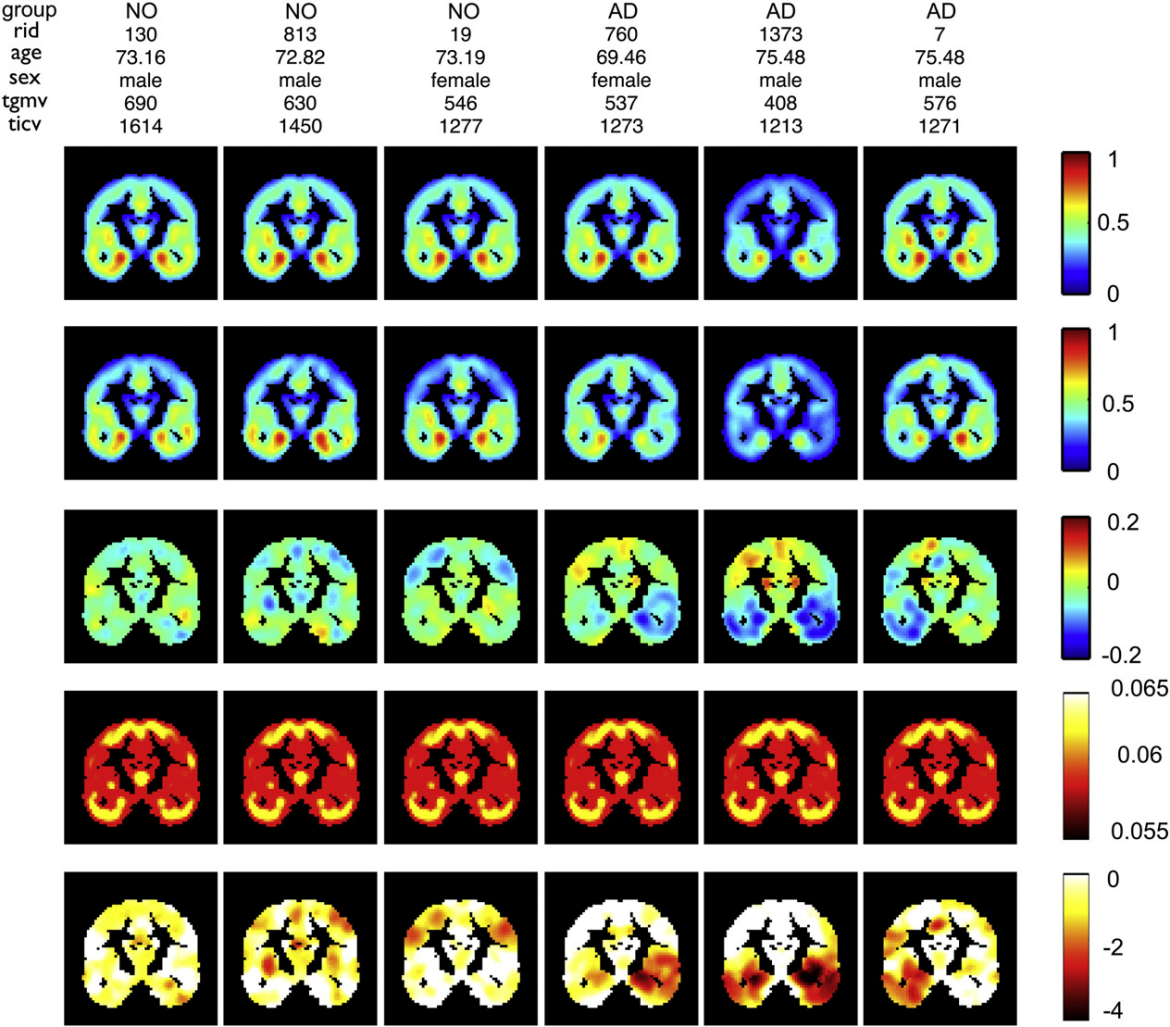
Model components and Normative Probability Maps (NPMs) for 3 NO and 3 AD subjects from the ADNI testing sample in coronal slice and 12 mm full width at half maximum (FWHM) data. Individual ADNI rids, age, sex, total gray matter volume (ml) and total intracranial volume (ml) are given. Descending rows show the prediction by the GP model, the observed local gray matter volumes, prediction errors (i.e. observed–expected values), the square root of predictive uncertainty and NPMs (i.e. local z-maps).

In order to validate the GP-based z-scores for single case inference in subjects with dementia, we also assessed global and local z-scores (i.e. NPMs) in subjects with clinical indications for neuropathology, in particular with diagnosis of Mild Cognitive Impairment and Alzheimer's disease. Fig. 8A shows ADNI test sample z-scores for local gray matter volume in the hippocampus voxel (24, − 12, − 18 MNI) and global brain volumes after GP model training with the full healthy aging database. The average z-scores across the healthy controls were found to be close to zero. In contrast, clinical group subjects' revealed decreased z-scores in hippocampus voxel volume and total gray matter volume and increased z-scores of total cerebrospinal fluid volume. Irrespective of the substantial variability in healthy aging, the z-scores of test patient's suggest additional local hippocampus and global gray matter volume atrophy. Assuming that the diagnosis of the ADNI subjects is true, one can compare the efficiency of local and global z-scores with the conventional approach of t-test based single case inference. For the particular purpose of comparison we applied Gaussian process classification to separate patients with pMCI and AD from controls only on the basis of the hippocampus voxel and the global volumes (Fig. 8B, see also Discussion). Fig. 8B middle column shows the receiver operating characteristic (ROC) curves and the area under curve (AUC) as performance metrics for the two dimensional classification using z-scores from hippocampus gray matter volume and one of the global brain parameters. Using local and global z-scores combined revealed a better classification performance in terms of AUC compared to the conventional t-value based separation on a subject by subject basis (Fig. 8B right column). Finally, in order to illustrate the proposed method, Fig. 9 summarizes model components and NPMs in 6 ADNI test subjects. Decreased z-scores are expected to emphasize local gray matter abnormalities due to atrophy or alternatively unknown covariate effects. Focal reductions of predictive probabilities were particularly observed in lateral and medial temporal lobe regions of many patients diagnosed with AD.

## Discussion

Here we applied Gaussian process models for prediction and single case inference about local and global brain structural abnormalities in aging subjects. We implemented a non-parametric generative model of healthy aging, which allows individualized predictions in patients at risk of developing dementia. Using simulations we demonstrated advantages of the approach over existing methods for the purpose of prediction and inference in healthy and diseased subjects. As a further proof of concept, we focussed on real MRI data in a large healthy aging VBM database and tested the GP models to detect abnormalities in the most common neurodegenerative disease, AD. An accumulating body of work has demonstrated that medial temporal lobe atrophy is a consistent and pathologically verified (Burton et al., 2008) marker for AD (for review see Frisoni et al., 2010) which also has been shown to have the strongest effect sizes in direct comparison of controls and AD (Risacher et al., 2010). Medial temporal atrophy is also one of the MR-based biomarkers discussed for revised definitions of AD (Dubois et al., 2010, McKhann et al., 2011). Applying our GP model to the test samples, we observed a considerable reduction of medial temporal lobe z-scores in patients diagnosed with MCI and AD.

As suggested by related studies using parametric models (Salmond et al., 2002, Scarpazza et al., 2013), we aimed at reducing potential biases due to violations of normality assumptions of modulated VBM data. As shown by Viviani et al. (2007), influences of non-normality can be successfully reduced by appropriate transformation of the data. Crucially, the deviations from normality do not follow a uniform pattern across voxels, and thus we applied a voxel specific Box–Cox transformation using a maximum likelihood method. By doing so, we observed a substantial improvement of residuals' normality, which was further increased by precedent application of 8 mm or 12 mm Gaussian smoothing kernels. We found that the local GP model evidence was strongly dominated by the variance of noise model, i.e. with lower residual variance resulting in higher evidence. Note, however that the observed evidence differences do not have the same meaning as in the context of Bayesian model comparisons (see e.g. Penny, 2012) where one compares different models of the same data rather than different models of different data, e.g. from different voxels. Most cortical regions provided slightly smaller noise variances compared to subcortical regions especially the thalamus and also the basal ganglia. These regional differences in the GP models might be due to effects of segmentation, nonlinear normalization, the total explained variance by the covariate space, and the true individual differences of local gray matter volume. There was a further tendency to a slightly smaller amount of noise in fronto-temporal compared to occipito-parietal gray matter regions which might be related to the fact that age-related effects in elderly subject brains are often found to be less emphasized in posterior brain regions (Fjell and Walhovd, 2010, Raz and Rodrigue, 2006).

Earlier studies demonstrated that VBM and parametric models afford inference about age-related gray matter volume differences in healthy aging groups (Good et al., 2001, Hutton et al., 2009, Kennedy et al., 2009, Ziegler et al., 2012b) and brain pathology in single patients (Colliot et al., 2006, Mehta et al., 2003, Mühlau et al., 2009, Salmond et al., 2003, Sehm et al., 2011). In addition, recent studies also showed the potential of recognition models and multivariate classifiers to decode early stage diagnosis based on brain scans in dementia and especially AD (Adaszewski et al., 2013, Davatzikos et al., 2009, Davatzikos et al., 2011, Klöppel et al., 2008, Misra et al., 2009, Westman et al., 2011, Westman et al., 2012). Although multivariate decoding models are expected to be powerful, sensitive, and highly accurate, we argue that using only ‘black box’ schemes might lack transparency and simplicity for decisions made in current clinical practice. Therefore, our approach aimed at decision support in the gap between multivariate classifiers (Klöppel et al., 2008) and qualitative visual inspection of scans (DeCarli et al., 2007, Korf et al., 2004).

A contribution of this paper is to apply a Bayesian approach that incorporates model- and predictive uncertainty for the single case inference. As recently emphasized by Klöppel et al. (2012), furnishing predictive probabilities in clinical disease state classification tasks can provide useful measures of the confidence for classification results. This idea was also explored by Marquand et al. (2010) using GP classification of whole brain patterns of brain activity in response to thermal pain (see also Hahn et al., 2011). We extend these results to GP regression of continuous variables and show the potential value of predictive probabilities to support clinician's decisions about gray matter abnormalities in aging subjects. We like to point out two interpretations. Firstly, the presented z-scores from the local NPMs and global indices aim to support clinical inference about single patients' brain structure at risk of dementia. In analogy to neuropsychological test scores, by inspecting the NPM and the three global z-scores of a patient, the clinician is quantitatively and transparently informed about the patient's brain volumes in relation to a large healthy reference sample. At the same time the approach accounts for the effects of important covariates and individual differences aging. Increasingly negative z-scores indicate an increased risk of local and global atrophy. Secondly, NPMs can be seen as part of a naive Bayesian inference, which clinical experts might follow when judging scans about alternative causes of atrophy in an individual patient at risk of developing dementia. Thus, to support inference about several causes, the NPMs of *P*(*scan*|*healthy*) might be complemented by specific disease likelihood maps, e.g. *P*(*scan*|*AD*) for Alzheimer's disease. The latter could be similarly obtained from local GP based pathology models in the clinical populations of interest.

As recently pointed out, multivariate recognition of AD disease states (Dukart et al., 2011) and voxelwise generative models of AD disease progression (Dukart et al., 2013) should necessarily account for regionally inhomogeneous age-related baseline changes in healthy controls. Thus, considering this variability our trajectory model presents an appropriate reference for detection of gray matter abnormalities in early and late disease onset, e.g. in early-onset vs. late-onset AD. Moreover, it extends existing approaches by avoiding the limitations of low degree polynomial expansions of age (see also Fjell et al., 2010). The shape of lower degree models is restrictive and imposes strong constraints on the unknown developmental process. This might reveal poor estimates of the structural trajectory in analyses spanning several decades of the lifespan. Although higher degree polynomials provide more flexibility of trajectory shape than lower degree polynomials, for our purpose of predictions we prefer a non-parametric GP covariance model which does not require additional model comparisons for the selection of different polynomial degrees. Note, that using a GP model has some formal correspondence to regularization problems using penalties on derivatives and also smoothing spline models (Wahba, 1990) can be seen as a special case of the GP framework (see e.g. Sections 6.2 and 6.3 in Rasmussen and Williams, 2006). In contrast to recent applications of smoothing splines and kernel estimators in structural neuroimaging, the GP framework applies Bayesian evidence based optimization of length scale parameters instead of using cross-validation procedures to specify the smoothness or kernel bandwidth parameters respectively. Note, that unlike polynomial models, a squared exponential GP covariance implements a local regression method, i.e. the local structural trajectory only depends on data points of subjects with similar ages. This is particularly useful for lifespan studies, where additional inclusion of older (younger) subjects might not be expected to change predictions for younger (older) participants respectively. Our approach has some similarity to outlier detections schemes, used for patient classification. In a recent study Mourao-Miranda et al. (2011) addressed the problem of measuring departures from a distribution of Gaussian multivariate patterns of fMRI activity by modeling the boundary of this distribution.

It is important to note that we aimed to provide a map to support expert decisions about the current state of atrophy rather than to predict the subject's disease status per se. In contrast, we here address inference at the level of local and global gray matter volumes. The above presented classification of subjects based on local and global volumes was used to provide a proof of principle that GP z-scores might afford slightly more accurate characterization of individuals compared to existing methods. Otherwise, for the purpose of inference about the causes of atrophy, e.g. AD vs. MCI, AD vs. NO, etc., the whole pattern of features combining the NPM and global z-scores is expected to be more informative than single voxels and can be feed into supervised learning algorithms. We would also like to mention the commonalities and differences with the BrainAGE approach which was recently introduced by Franke et al. (2010). The author's multivariate age decoding scheme has shown potential applications for accurate predictions of conversion of MCI to AD (Gaser et al., 2013). Both models, BrainAGE and NPMs exploit prediction errors under the assumption of a model of healthy brain aging and take advantage of the increased availability of healthy subject MRI data. However, the approaches also fundamentally differ with respect to the level of inference and treatment of individual differences. BrainAGE provides a whole brain pattern-based index of age-typical atrophy, whereas NPMs quantify normative expectation and confidence about local gray matter volumes. We therefore argue, that both approaches provide complementary and potentially useful information about a single elderly patient's brain. In contrast to the application of multivariate classifiers (for review Klöppel et al., 2012) the integration of likelihood and priors is still performed by a clinician himself. The benefit of this approach though can only be verified in clinical settings using this technique in direct comparison with visual inspection of structural MRI scans in native space. Notably, native space T1 scan inspection provides qualitative in contrast to quantitative information about the atrophy in single cases. Moreover, the prior knowledge accumulated over years of clinical experience is still expected to reflect smaller sample sizes than the 1238 subjects. However, at the moment we can only speculate that clinicians who additionally inspect the quantitative model based NPMs and three global z-scores might come to more valid clinical conclusions facing patients with different brain sizes, sex, and up to five decades of age differences. Further validation studies might address the comparison of qualitative and quantitative single case inference schemes in clinical settings.

In order to facilitate applications in other samples, we explored the effects of image smoothness and sample size for our GP model and individualized predictions, especially the predictive uncertainty. Using large smoothing kernels for VBM data, a higher validity of statistical tests is achieved at the expense of the fine-grained structure of the cortical mantle (Viviani et al., 2007). We observed that stronger smoothing consistently reduced the local and the whole brain average GP model noise variance. The noise level has a substantial contribution to the variance of the predictive distribution. Thereby, predicting local gray matter volume in single subjects of the ADNI test data, we observed that stronger smoothing reduced uncertainty. Note, by using z-scores of the predictive distribution to obtain the NPMs, the uncertainty differences are expected to affect the sensitivity to detect local abnormalities. For instance, larger absolute gray matter reductions would be necessary to produce the same z-score reductions (in the NPM) within two voxels with high compared to low predictive uncertainty. Although this might introduce differences in the NPMs across different voxels, this is a desired behavior of the model and renders inferences about local gray matter volumes more valid. Regarding the question of a sufficient adult control sample size for valid predictions in test subjects, we observed more consistent hyperparameters and latent variable estimates by training with larger subsamples of our database. Similar to parametric models, larger random training subsamples were also found to better capture potential late-life nonlinearities and accelerated structural decline (Fjell et al., 2012, Ziegler et al., 2012b), which then affords more specific predictions in subjects at risk for developing dementia. Note, that using the presented covariance kernel forms an information bottleneck that compresses individual differences in the covariate space to one kernel matrix for subsequent predictions. Consequently, using larger training samples also results in more densely sampled covariate spaces which afford more precise individualized predictions. Inspecting test subjects' hippocampus predictions using a training database of increasing size, we found a rather constant average uncertainty of predictions but substantially reduced fluctuation of precision across individual test subjects. According to this analysis, a larger sample size favors a higher stability of the predictive uncertainty which is expected to result in a more constant sensitivity to detect brain abnormalities across different individuals, e.g. with different ages, brain sizes, etc. Note, however, that due to the domination of the noise term in the predictive uncertainty, the effects of different degrees of smoothing and variability across the cortical mantle, is expected to be more substantial than fluctuations due to individual differences in very large samples.

Some caveats and directions for development of the presented approach have to be mentioned. Firstly, structural brain aging is expected to be a highly individual process embedded in a complex and reciprocally interacting system including the genes, physiology, behavior, and the individual cultural environment (Baltes et al., 2006, Jagust, 2009). We assume that this process becomes manifest in the individual brain trajectories forming a flowfield of lifespan brain development after accounting for subject's covariates. This is in line with recent evidence from mixed-effects analysis of repeated measures MRI, showing substantial individual differences of regional structural trajectories (Raz et al., 2010). Thereby, defining a sufficient normative reference for pathological structural aging, one necessarily requires an approximation of the structure of the flowfield including the most common individual differences. Apart from potential secular trends and cohort effects of a purely cross-sectional design (for a more detailed discussion of this point see Ziegler et al., 2012a) we here make the strong assumption that the hidden causes of individual differences in elderly are fully captured by the above considered covariate space D. As recently pointed out by Doyle et al. (2013b), personalized modeling approaches are required in order to make personalized medicine reality. We might speculate that using a sufficiently high-dimensional multivariate parametrization of individual differences including genes, education, cognitive scores (see e.g. Ziegler et al., 2013), and behavior, the considered cross-sectional trajectory estimates might converge to estimates from repeated measures MRI design. Otherwise, combining cross-sectional with high quality longitudinal segmentations (Jovicich et al., 2013, see e.g.) might further improve model-based predictions and inference.

Secondly, a limitation of this study is the potential adverse effects of pooling across MRI scanners and sequences (Jovicich et al., 2013, see e.g.). However, the purpose of the proposed database-based prediction and inference in elderly subjects is to aim at generalization to new clinical scanners while exploiting the benefits of a large healthy aging reference sample and the diverse appearance of normal aging. In this particular model, local and global scanner effects are expected to be captured by increases of the model error variance. Within the Bayesian inference framework, we expect this to result in reduced predictive confidence for particular brain regions with expectable high scanner related artifacts, e.g. in subcortical regions. The proposed z-scores account for this local increase of uncertainty in terms of higher deviation from normality to be required to observe the same z-score. Thus, the gains of generalization to new clinical scanners come at the cost of a reduced sensitivity for gray matter abnormality detection. Notably, an alternative model accounting for all site effects is likely to result in biased conclusions in predictions on new scanners due to severe overconfidence. We argue that the provided comparisons of GP-based z-scores in clinical groups still demonstrate a potentially useful characterization of unseen subjects from new scanners. Future studies might focus on generalizable assumptions about forms of scanner related variability which could be included in model training and inform predictions on scanners with specific imaging parameters.

Thirdly, a serious problem with GP methods is that it requires computation which grows as *O*(*n*^3^), where n is the number of subjects. This is computationally expensive, especially if we aim at the advantages of large sample healthy aging databases. Future studies might explore sparse approximation techniques (Quiñonero-Candela and Rasmussen, 2005, Quinonero-Candela et al., 2007) for more effective local models or spatial regularization (see e.g. Banerjee et al., 2008, Sang and Huang, 2012).

Finally, the local brain morphology in our GP models was restricted to gray matter segments obtained from VBM. Although medial temporal lobe atrophy is one of the most-established imaging biomarkers for AD (Frisoni et al., 2010, Teipel et al., 2013), our GP framework might be extended to other potential disease sensitive sequences and modalities, e.g. local white matter hyperintensities (Brickman et al., 2012, Carmichael et al., 2010) or [18F]fluorodeoxyglucose Positron Emission Tomography (FDG-PET) (Dukart et al., 2013) as well as amyloid PET (Quigley et al., 2011).

## Conclusion

We argue, that decisions about subjects at risk to convert to pathological aging might be supported via transparent evidence given the quantitative models of normal and pathological aging. Normative probability maps and global brain volume z-scores afford individualized detection of abnormalities and appropriately account for the uncertainty of the model and the model's predictions due to random influences, e.g. noisy observations and sampling.

## Footnotes

2 Data used in the preparation of this article were obtained from the Alzheimer's Disease Neuroimaging Initiative (ADNI) database (adni.loni.ucla.edu). The ADNI was launched in 2003 by the National Institute on Aging (NIA), the National Institute of Biomedical Imaging and Bioengineering (NIBIB), the Food and Drug Administration (FDA), private pharmaceutical companies and non-profit organizations, as a $60 million, 5-year public–private partnership. The primary goal of ADNI has been to test whether serial magnetic resonance imaging (MRI), positron emission tomography (PET), other biological markers, and clinical and neuropsychological assessment can be combined to measure the progression of mild cognitive impairment (MCI) and early AD. Determination of sensitive and specific markers of very early AD progression is intended to aid researchers and clinicians to develop new treatments and monitor their effectiveness, as well as lessen the time and cost of clinical trials. The Principal Investigator of this initiative is Michael W. Weiner, MD, VA Medical Center and University of California-San Francisco. ADNI is the result of efforts of many co-investigators from a broad range of academic institutions and private corporations, and subjects have been recruited from over 50 sites across the U.S. and Canada. The initial goal of ADNI was to recruit 800 adults, ages 55 to 90, to participate in the research, approximately 200 cognitively normal older individuals to be followed for 3 years, 400 people with MCI to be followed for 3 years and 200 people with early AD to be followed for 2 years. For up-to-date information, see www.adni-info.org.
